# Permanent dry soil layer a critical control on soil desiccation on China’s Loess Plateau

**DOI:** 10.1038/s41598-019-38922-y

**Published:** 2019-03-01

**Authors:** Chunlei Zhao, Xiaoxu Jia, Kate Gongadze, Ming’an Shao, Lianhai Wu, Yuanjun Zhu

**Affiliations:** 1 Key Laboratory of Ecosystem Network Observation and Modeling, Institute of Geographical Sciences and Natural Resources Research, Chinese Academy of Sciences, Beijing, 100101 China; 2 State Key Laboratory of Soil Erosion and Dryland Farming on the Loess Plateau, Northwest Agriculture & Forestry University, Yangling, 712100 China; 3 College of Resources and Environment, University of Chinese Academy of Sciences, Beijing, 100190 China; 4 Rothamsted Research, North Wyke, Okehampton, Devon, EX20 2SB UK

## Abstract

The wide spread of dry soil layers (DSL) in China’s Loess Plateau region has negative effects on the ecosystem, including soil degradation and vegetation failure. To understand the temporal persistence of DSL, a ca. 860 km south-north transect was established and soil water content of the 0–5 m depth soil layer repeatedly measured for a period of four years. The results indicated that DSL varied with time and had a distribution area over 21.5–47.0% in the 860 km transect during the study period. The DSL could be divided into temporary and permanent types based on the length of period for which the soil remains dry. While temporary DSL is recoverable, permanent DSL (which existed in 47 out of 86 sites) was apparently unrecoverable as it persisted throughout the observation period. Permanent DSL was characterized by high temporal persistence, severe soil desiccation and thick dry layers; all of which suggested severe negative effect on the ecosystem. Non-climatic factors, rather than climate factors, contributed more to the formation of permanent DSL in the study area. Thus, it was suggested that policies and measures should be enacted to control especially permanent DSL formation in the region.

## Introduction

The formation of dry soil layer (DSL) is a global hydrological process that has been reported in China^1^, Russia^2^, Eastern Amazonia^3^ and Southern Australia^4^. It generally occurs in arid, semi-arid and semi-humid regions, mainly due to excessive depletion of deep soil water by vegetation, evapotranspiration and scarce precipitation recharge over a long time^5,6^. DSL is defined as a soil layer with soil water content (SWC) less than the stable soil field capacity^7–9^. Stable soil field capacity is generally defined SWC at 60% of the field capacity^6,10^. DSL can hamper water movement and exchange in the soil profile and can cause severe eco-hydrological problems. This is a common occurrence in China’s Loess Plateau (CLP) region, where frequent water shortage and high soil erosion has made the ecosystem highly fragile^6,11,12^. DSL was first discovered in the 1960s in the semi-arid region of CLP^1^, but was given little attention until the 1980s. Since then, DSL has been widely reported in croplands, grasslands and forestlands across CLP as efforts to increase crop yields and large-scale re-vegetation have, but all worsened soil water conditions in the region^5,6,11^.

The negative impacts of DSL on soil hydrological processes such as water movement and on the ecosystem are well in recent literature^5,7^. DSL prevents vertical water exchange along the soil profile, breaking water cycle in the soil-plant-atmosphere continuum^5,11^ and threatening the sustainability of the environment^13,14^. With sufficient soil water, the thick loess deposit (92 m on average) in CLP can support strong vegetation growth^15^. The support function of the soil has reduced drastically with the development of DSL, reducing water availability to support vegetation^14^. The persistence of DSL for a long period can cause vegetation destruction, land degradation and loss of ecosystem^16,17^.

Studies have discussed in significant details the definition, spatial variability and factors of DSL in CLP^5,8,18^. DSL generally varies in space due to spatial heterogeneity of soil, land use, climate and landform^11^. Wang *et al*.^6^ noted that DSL is dominated by precipitation and soil type at regional scale and that it is generally thick (170–220 cm) in the western and central regions of the plateau. Then at small spatial scale (watershed or slope scale), the formation DSL is correlated with the type and age of vegetation^18,19^. DSL also varies over time due to temporal variations in plant water uptake. Li *et al*.^20^ investigated temporal dynamics of DSL in a rainfed wheat field and discovered that it generally occurs in April and recovers in February the next year. However, for *Medicago sativa L*. land in the northwest of CLP, DSL hardly recovers under precipitation and therefore persists for several years^21^. Based on the temporal persistence of DSL, it could be divided into two types: the temporary and the permanent DSL^7,11^. Permanent DSL usually develops in arid and semi-arid regions where there is low precipitation and high evapotranspiration. Then temporary DSL mostly develops in dry seasons or during years of low precipitation in semi-humid regions^11,22^. Soil water can be replenished in temporary DSL conditions by precipitation^22,23^.

In 1999, restore vegetation started under the “Grain-for-Green” initiative by planting trees or grasses on the plateau^24^. The large-scale revegetation with exotic plant species uses excessive deep soil water due to the rooting depth of the plants^25^. Moreover, global warming is predicted to continue going into the future, which will increase evapotranspiration due to increased plant water uptake^26,27^. Hence, the vegetation drive and climate change will further intensify soil desiccation in the region, expanding further to area under DSL. Studies on DSL in CLP have most been from short-term field observations. In fact, long-term data on DSL can comprehensively capture the information of such soil layers. Therefore, there is the felt need to strengthen investigation of the dynamics of DSL and the influencing factors under long-term observation. This is critical for sustainable use of soil and water resources and for successful vegetation of CLP.

**Figure 1:**
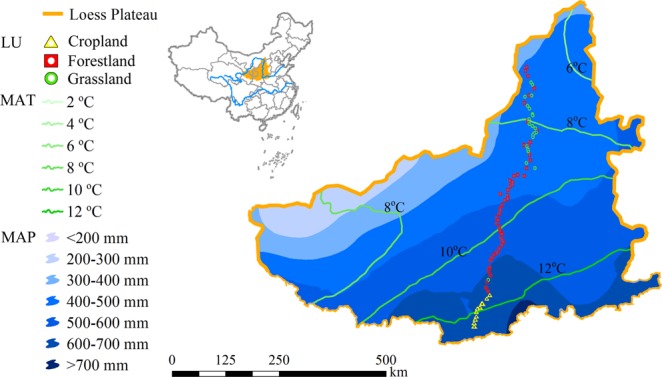
Map depicting the 86 sampling sites across the land use (LU) types, mean annual temperature (MAT, °C) and precipitation (MAP, mm) across China’s Loess Plateau.

**Figure 2:**
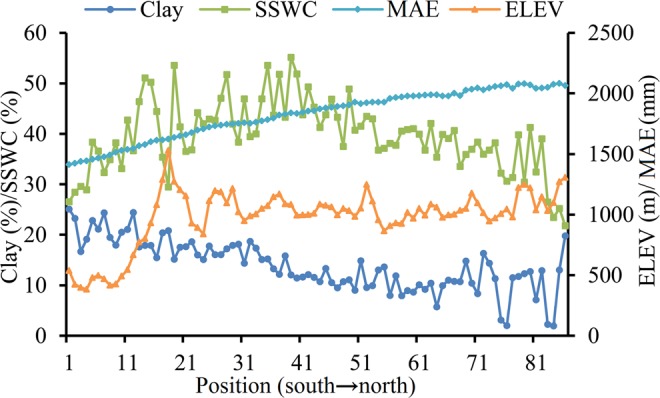
Plots of the dynamics of clay content (Clay, %), saturated soil water content (SSWC, %), elevation (ELEV, m) and mean annual evaporation (MAE, mm) along the south-north transect of China’s Loess Plateau.

In this study, a long-term (2013–2016) observation of SWC along with 12 environmental factors were used to analyze the temporal characteristics and driving factors of DSL across a south-north transect of CLP (Figs 1 and 2). The objectives of this study were to: (1) analyze temporal persistence of DSL over the long term; and (2) address the contributions of external factors to the development of temporary and permanent DSL.

## Results

### Space-time fabric of DSL

**Figure 3:**
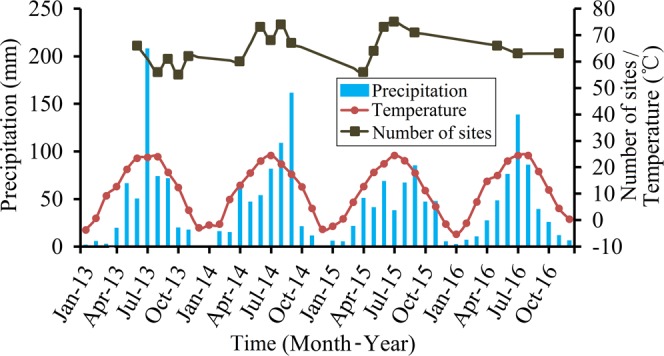
Plots of the dynamics of monthly precipitation, mean monthly air temperature and the number of sites with dry soil layer (DSL) along the transect of China’s Loess Plateau for the 2013–2016 study period.

**Figure 4:**
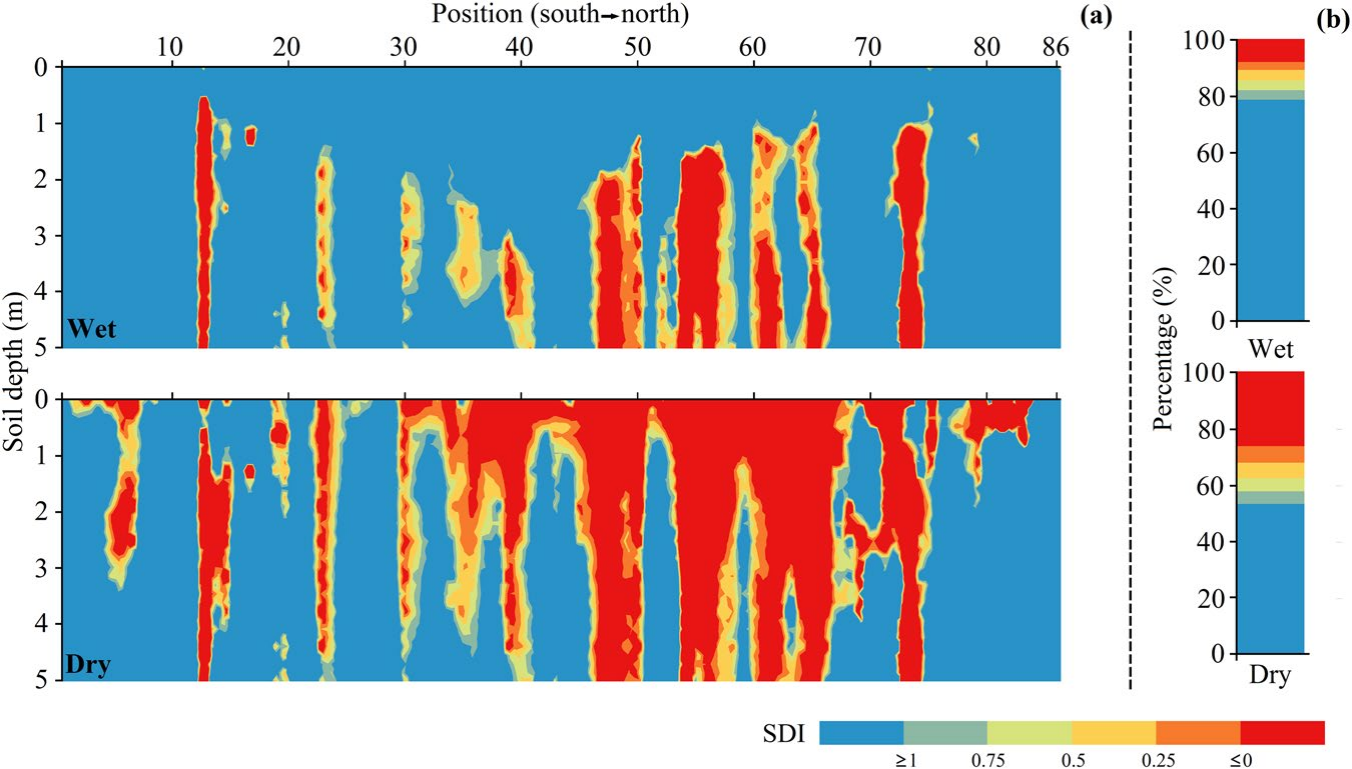
Plots of the dynamics of soil desiccation index at various soil depths in August 2013 (wet) and July 2015 (dry) (**a**), and the graduated scale bar depicting the degree of soil desiccation index (**b**).

Sites with DSL during the observational period are plotted in Fig. 3. Out of the 86 sites of SWC measurement, an average of 76% had DSL. DSL was most prevalent in July 2015 and had the least occurrence in August 2013. The two extreme states of the soil in terms of DSL were grouped under dry and wet conditions, respectively. The distributions of soil desiccation index (SDI) at various soil depths under the two conditions are plotted in Fig. 4a. While under the wet condition, only 21.5% of the area in the transect had DSL, this proportion increased to 47.0% under the dry condition. For both conditions, DSL mainly occurred in the central region of the transect. This included the sites occurring between 44 and 65 in the south-north direction. For sites 2–8 and 78–83, DSLs were relatively weak, with thin thickness (DSL-T < 3 m) and shallow depth (DSL-F <0.4 m). For these sites, DSL can recover during the wet season. Composition of the degrees of soil desiccation are shown in Fig. 4b. Under dry conditions, DSLs exhibiting extreme desiccation accounted for over 50% of the areas with DSL. Under wet conditions, soil water condition along the transect generally improved, with significant drop in the area with extreme desiccation (Fig. 4b).

### Temporary characteristics of DSL

**Figure 5:**
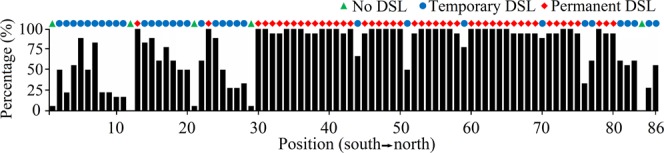
The temporal occurrence percent dry soil layer (DSL) over the 18 measurements at each sampling site.

The frequency of occurrence of DSL at each site during the experimental period is shown in Fig. 5. Based on the defining of temporary and permanent DSL, 34 sites had temporary DSL and 47 sites permanent DSL. For sites with temporary DSL, the average frequency of occurrence of DSL was 9, indicating that it occurred for 50% of the year. However, for sites with permanent DSL, it existed almost throughout the year. Permanent DSL mainly existed in the central and north-central regions of the transect, but and were scattered in the south central of CLP (Fig. 5).

**Figure 6:**
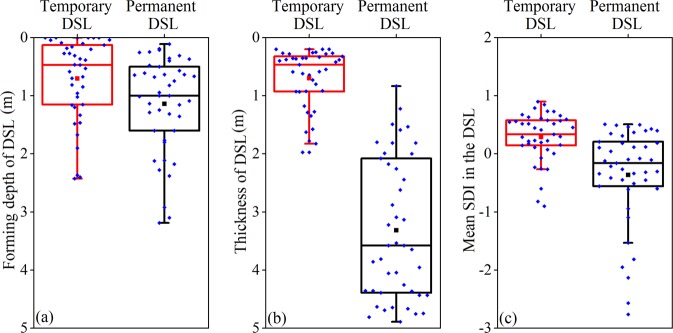
Box and whisker diagrams showing the range, mean and the 25th, 50th and 75th percentiles of temporary and permanent dry soil layer (DSL) indexes in the study area.

DSL-F was generally shallower under temporary than under permanent DSL, with respective means of 0.7 m and 1.1 m (Fig. 6a). Average DSL-T under permanent (3.3 m) was nearly 5 times that under temporary DSL (0.7 m). Moreover, about 50% of DSL-T was more than 3.6 m under permanent DSL, with some up to 4.8 m deep (Fig. 6b). Furthermore, mean DSL-SDI under temporary (0.3) was generally higher than that under permanent (−0.4) DSL. The average degree of desiccation under permanent DSL was classed as extreme, with over 50% SDI under permanent DSL less than zero (Fig. 6c).

### Factors affecting temporary and permanent DSL

The results off redundancy analysis are shown in Table 1. Both climatic (precipitation, evaporation, temperature and aridity index) and non-climatic (NDVI, saturated soil water content, soil clay content and soil bulk density) factors were responsible for temporal persistence of DSL. However, permanent DSL was mainly controlled by non-climatic factors such as land use, elevation, saturated soil water content and soil bulk density.

**Table 1:** Results of forward selection and Monte Carlo permutation for redundancy analysis of temporary and permanent dry soil layers (DSLs).

Factors	Variables	R^2^	*p*
Temporal DSL			
Climatic factors	MAP	0.219	0.004
	MAT	0.241	0.003
	MAE	0.183	0.014
	MAI	0.215	0.005
Non-climatic factors	LU	0.126	0.072
	NDVI	0.289	0.002
	ELEV	0.033	0.514
	Aspect	0.011	0.816
	Gradient	0.018	0.718
	SSWC	0.172	0.029
	Clay	0.318	0.001
	BD	0.154	0.042
Permanent DSL			
Climatic factors	MAP	0.008	0.843
	MAT	0.047	0.354
	MAE	0.010	0.816
	MAI	0.017	0.699
Non-climatic factors	LU	0.133	0.044
	NDVI	0.090	0.141
	ELEV	0.161	0.034
	Aspect	0.023	0.603
	Gradient	0.103	0.115
	SSWC	0.153	0.035
	Clay	0.051	0.357
	BD	0.193	0.011

Mean annual precipitation (MAP, mm); mean annual evaporation (MAE, mm); mean annual temperature (MAT, °C); mean annual aridity index (MAI); land use (LU); elevation (ELEV, m); slope aspect (Aspect, °); slope gradient (Gradient, °); saturated soil water content (SSWC); clay content (Clay, %); bulk density (BD, g/cm^3^).

**Figure 7:**
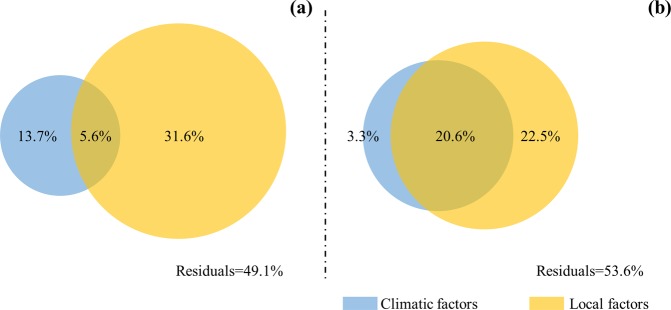
Results of the variations in partitioning analysis used to assess the relative contribution of climatic and non-climatc factors driving the characteristics of temporary (**a**) and permanent (**b**) dry soil layers (DSLs).

Redundancy analysis showed that 50.9% of total variability of temporary DSL and 46.4% of that in permanent DSL was explained by the 12 factors. The environmental factors contributed greatly to temporal persistence of DSL (Fig. 7). Both climatic (13.7%) and non-climatic (31.6%) factors contributed variously to temporary DSL. However, non-climatic factors had the highest contribution, suggesting that these factors played a more important role in temporal variations of temporary DSL. The interaction between climatic and non-climatic factors accounted for 5.6% of the variation of temporary DSL (Fig. 7a). Similarly, non-climatic factors accounted for the largest (22.5%) variation of permanent DSL. Also, the interaction between climatic and non-climatic factors contributed more (20.6%) to variation of permanent DSL than that of climatic factors (3.3%) alone (Fig. 7b). This suggested that temporal variation of DSL was driven both by climatic and non-climatic factors, but that the latter contributed more.

## Discussions

### Long-term dynamics of DSL

Studies show that DSL has a high spatial heterogeneity in CLP region^6,11,14^. However, we found that DSL also has a strong temporal heterogeneity along the south-north transect of CLP. In this study, 40% of the sites (34 out of 86 sites) had temporary DSL (Fig. 5). DSL in at these sites had occasional occurrences (only 9 of 18 measurements). This feature of DSL indicated that short-term observation was not sufficient for a clear insight into the dynamics of DSL, which is useful for classification and management of DSL. Over the four years of measurements in this study, the area with the minimum DSL distribution along transect was about half of the maximum value. Thus data from DSL studies based on short-term observations were, to some extent, unrepresentative (Fig. 4a). Long-term observations of DSL was needed to capture the full picture of the variability of DSL the study area.

### Factors and patterns of DSL

The temporary DSL in the soil profile was shallower and thinner than that permanent DSL, contributing to the lower frequency of occurrence of temporary DSLs (Fig. 6). In CLP, infiltration depth of precipitation is usually less than 2 m^28^. Continuous climatological drought and excessive vegetation water use could worsen desiccation of the upper 2 m soil layer. However, soil water in this layer can also be easily replenished by precipitation in the rainy season, making the occurrence of DSL temporary.

The spatial distribution of permanent DSL was closely related to local environmental conditions including low precipitation and high evapotranspiration, common phenomena in arid and semi-arid regions^22^. Soil water replenishment is closely related with precipitation and infiltration capacity of the soil^9^. For the sites in the central north of the transect, low precipitation (compared with the southern part of the transect) and low soil infiltration capacity (compared with the northern part of the transect) hindered soil water replenishment (Fig. 2); explaining the wide spread of DSL in the region. The frequency of occurrence of DSL decreased in the north of the transect (Fig. 5). This could be attributed to low clay content (hence high infiltration capacity) and the vegetation effect (low grass water uptake) in the region (Fig. 2). This agreed with the report that the DSLs in grasslands and croplands are generally thinner than in forestlands^6^.

The correlations between DSL characteristics and different tested parameters have been analyzed in many other studies^6,11,13^. However, most of these of these studies overlook the formation mechanisms of DSLs, making it difficult to determine the factors driving the formation of DSLs. Here, we used collected field data and multivariate analysis to group DSLs into temporary and permanent formations, from where the driving factors determined. Our results suggested that the dominant factors driving the formation of temporary and permanent DSLs were different. The role of non-climatic factors was crucial in both bases of DSL formation. Compare with permanent DSL, however, climatic factors contributed more to the variation in temporary DSL (Table 1 and Fig. 7). The upper soil layers were more sensitive to short-term changes in climate^29,30^. Fluctuation of SWC in the deep soil layers was mitigated by the insulating effect of the top soil layers. In the study, 75% of DSL-F and DSL-T under temporary DSL were less than 1.1 m and 0.9 m, respectively, which were within the depth of precipitation recharge. Thus, temporary DSL could be a less concern when making policies for managing DSL conditions.

### Permanent DSL critical in CLP

Classification of DSLs provides the framework for in-depth understanding of the current status of soil water condition in the Loess Plateau. In terms of the recovery capacity of temporary DSL and the related negative effects of permanent DSL on the soil hydrology, the focus should be on regulation policies and control measures mainly for permanent DSL.

Climatic factors have significant influence on temporary DSL and there were critical in preventing temporary DSL conditions. In converting croplands to forestlands, planting densities or biomass used should be based on soil water capacity^31^. Also non-climatic factors were critical for permanent DSL conditions. Thus measures related to non-climatic factors including land use conversion, vegetation selection (native species that are drought tolerant and less water consuming) and slope terracing are key in preventing permanent DSL formation. For instance, the vegetation reconstruction adopted in the “return-to-farmland” drive should be based on natural vegetation succession in the region. Here, *Pinus tabulaeformis* Carr. Can be used as afforestation species and/or fish-scale pits or terraced fields constructed on slopes^22,24,28,32^. Unsuitable measures such as planting trees in high densities and introducing exotic tree species could result in high use of soil water due to deep reach of the root system and high water extraction capability^5,9^. That is reason for the wide spread of DSLs in forestlands in the study area. Studies show that DSLs exist in 82% of the forestlands in the plateau^23^. This study also noted a similar distribution of DSL conditions in the forestlands in the region, the main land use type with spreading permanent DSL conditions (Fig. 4).

## Conclusions

In this study, we used 860 km south-north transect in CLP to investigate temporal persistence of DSL. The results suggested that long-term observation was required for full knowledge of DSL dynamics in the study area. It was more reasonable to divide DSLs into temporary and permanent conditions in order to determine the formation and control processes. Permanent DSL was mainly spread across the central and north-central parts of CLP. Here, more attention was needed due to low precipitation recharge and severe negative effects on the ecosystem. Efforts to control DSL should focus on areas with permanent DSL in order to prevent any such negative effects. Non-climatic factors (e.g., soil texture, micro-topography and soil hydraulic properties) were the dominant driving factors off permanent DSL and therefore critical for DSL recovery and prevention in the study area.

## Materials and Methods

### Study area

China’s Loess Plateau (CLP, 34°–45°5′N, 101°–114°33′E) has an area of 62 × 10^4^ km^2^ (Fig. 1) and elevation range of 200–3000 m. The plateau is covered by loess deposit that is on average 50–200 m thickness. The terrain of the plateau could be described as a typical Yuan (a large flat surface with little or no erosion) and Mao (an oval or round loess hill), Liang (a long narrow range of hills), with various forms of hills and gullies^33^. It has arid and semi-arid continental monsoon climate, with an annual evaporation of 1400–2000 mm^24^ and mean annual precipitation of 150–800 mm from the northwest to the southeast of the plateau. About 55–78% of precipitation occurs during the period from June to September. The mean annual temperature is 3.6 °C in the northwest and 14.3 °C in the southeast (Figs 1, 2). The main soil types from south to north include Haplic Luvisols, Terric Anthrosols, Calcic Chernozems, Aridic Leptosols, Calcaric Regosols, Calcic Kastanozems and Aridic Arenosols^32,34^. The vegetation along the southeast-northwest transect changes from forest to forest steppe, to typical steppe, to desert steppe and then to steppe desert.

### Site selection

The SWC measurements were done along a *ca*. 860 km south-north transect in CLP (Fig. 1). Alone the transect from south to north, the land use types include cropland, forestland and grassland. The cropland is usually cultivated with winter wheat and summer maize and is not irrigated. The main tree species in the forestlands are black locust (*Robiniapseudoacacia L*.), korshinsk peashrub (*Caraganakorshinskii*), apple (Maluspumila), apricot (*Armeniacasibirica L*.) and jujube (*Zizyphusjujuba*), all of which are artificial forestlands. The grasslands are both artificial and natural, and mainly comprised of *Medicago sativa L*., *Stipa bungeana* and *Lespedeza davurica*. The interval between SWC sampling sites was 10 km and 86 sampling sites were covered along the transect (Fig. 1). The basis of the site selection included: (1) representation of main land use, topography, soil type and vegetation type in a range of vision; (2) relative homogeneity of soil type and landform, etc.; (3) farness from lowland area to avoid water table effect; and (4) at least 200 m away from road to avoid human disturbance.

### Data collection

An aluminum-made neutron tube (5.2 m in length and 0.06 m in diameter) was installed at each site. We used a CNC503DR neutron probe (Beijing Super Power Company, Beijing, China) to obtain 0–5 m neutron readings (*CR*) at an interval of 0.2 m. Then, *CR* was converted into soil water content (*θ*, volumetric, %) by:

1 $$\theta =0.5891\times CR+0.0089$$

We used 18 times of SWC measurements during 2013–2016. Each measurement campaign (all 86 sites covered in every visit) took 3–4 days. At each site, 0–5 m soil samples were taken using a 5 cm diameter soil auger at an interval of 1 m. The soil samples were air-dried and passed through 2 mm mesh sieves for laboratory analyses. Clay content (Clay, %, USDA taxonomy) was determined by the laser diffraction method (Mastersizer 2000, Malvern Instruments, Malvern, England)^35^. A 0.4 m deep pit was excavated to obtain undisturbed soil samples at the 0–0.2 and 0.2–0.4 cm soil layers using a cutting ring (volume of 100 cm^3^ and height of 5 cm) at each site. For each undisturbed sample, soil water retention curve was determined using the centrifugation method (HITACHI CR21G centrifuge; 20 °C)^36^. Field capacity (at a suction of −0.03 MPa) and wilting soil water content (at a suction of −1.5 MPa) of each sample were derived from the curve^37^. Saturated soil water content (SSWC, %, volumetric) was determined from the loss of mass in the undisturbed samples during the period from saturation to dryness in 24 hours and at 105 °C. Bulk density (BD, g/cm^3^) was determined based on the mass of the soil core after 24 hours drying and the volume of the cutting ring. The mean saturated soil water content and bulk density in the 0–0.2 and 0.2–0.4 m soil layers were calculated as the surface saturated soil water content and bulk density for each sampling site. The RTK-GPS receiver (5 m resolution) was used to record the geographic coordinates and elevation (ELEV, m). Slope gradient (Gradient, °) and slope aspect (Aspect, °) were measured using a geological compass. The mean normalized differential vegetation index (NDVI) of 18 sampling occasions calculated for each sampling site was considered representative of the coverage and growth of the vegetation. The NDVI data were derived from MOD09GQ products of Moderate Resolution Imaging Spectroradiometer (MODIS; https://lpdaac.usgs.gov), which provided daily surface reflectance and NDVI datasets. Monthly climatic data for 2012–2016 (precipitation, temperature, evaporation and aridity index) from 73 weather stations in CLP were downloaded from China Meteorological Data Sharing Service System (http://cdc.cma.gov.cn/). The mean annual precipitation (MAP, mm), temperature (MAT, °C), evaporation (MAE, mm) and aridity index (MAI, ratio of evaporation to precipitation) at each station were calculated from the monthly climatic data. Then the climatic data for each site were obtained through universal kriging (100 × 100 m resolution) interpolation.

### Definition of DSL and evaluation index

In this study, soil desiccation index (*SDI*) parameter was calculated to evaluate the state of soil water in a given soil layer. *SDI* is defined as the ratio of the soil water to total available soil water in a DSL for a given soil type^19^:

2 $$SDI=\frac{SWC-WSWC}{SFC-WSWC}$$

where *SWC* is the soil water content at the time of measurement (volumetric, %); *WSWC* is the wilting soil water content (volumetric, %); and *SFC* is the stable field capacity (volumetric, %). *SDI* is generally divided into six levels^19^ — (a) extreme if *SDI* <0; (b) strong if 0 ≤ *SDI* <0.25; (c) severe if 0.25≤ *SDI* <0.5; (d) medium if 0.5≤ *SDI* <0.75; (e) slight if 0.75≤ *SDI* <1 and (f) normal if *SDI* ≥1.

Due to limited precipitation and lack of water resources in CLP, soil water content in this region is below field capacity for most of the time, and is usually within 50–70% of the field capacity. This water deficiency is a ubiquitous normal phenomenon in the plateau, which researchers define as stable field capacity^7,11,22^. In practice, the researchers find that 60% of the field capacity can be used as the stable field capacity for the loess in CLP^13,14,18^. Thus, soil layer with SWC less than stable field capacity was classed as DSL.

Based on the definition of DSL and the calculation of *SDI*^6,8,38^, SDI <1 indicated the occurrence of DSL. Three indexes were used to characterize DSL in this study: (1) DSL forming depth (DSL-F) — i.e., the depth at which DSL starts; (2) DSL thickness (DSL-T) and (3) mean *SDI* within DSL (DSL-SDI) (Fig. S1).

### Definition of temporary and permanent DSL

We classified DSL based on the frequency of occurrence of DSL. Permanent DSL is defined as DSL at a site with >95% possibility of occurrence. Then temporary DSL at a site is defined as DSL with <95% possibility of occurrence. If the possibility of occurrence of DSL is <5%, it is assumed not to exist at the site.

### Statistical analysis

Spatial interpolation and mapping were done in ArcGIS 10.2 (ESRI® ArcMap™). The relationships between DSL indexes and environmental factors were derived from redundancy analysis in “Vegan” package, version R 3.3.1 (http://cran.r-project.org/web/packages/vegan). Statistical significance in redundancy analysis was determined by forward selection and Monte Carlo permutation test. The relative roles of climatic and non-climatic factors in affecting DSL were assessed using partial redundancy analysis. To remove the effects of different scales of data, the data for measured factors were standardized to zero mean and unit variance analysis further performed. The specific standardized equation is:

3 $${x^{\prime} }_{i}=\frac{{x}_{i}-\bar{x}}{{\sigma }_{x}}$$

where, $x{\text{'}}_{i}$ is the standardized *x*_*i*_; $\bar{x}$ is the standard deviation of factor *x*.

## Supplementary information

FigureS1

## Data Availability

The datasets generated during and/or analyzed during this study are available from the corresponding author on justifiable request.
