# Supplementary material for: Permanent dry soil layer a critical control on soil desiccation on China’s Loess Plateau
Source: Sci Rep. 2019 Mar 1;9:3296. doi: 10.1038/s41598-019-38922-y (PMC6397324; doi:10.1038/s41598-019-38922-y)
Supplement: Supplementary file 1 — FigureS1 [file 41598_2019_38922_MOESM1_ESM.docx]

**Permanent dry soil layer a critical control on soil desiccation on China’s Loess Plateau**

Chunlei Zhao^1^, Xiaoxu Jia^1,3^, Kate Gongadze^4^, Ming’an Shao^1,2,3^, Lianhai Wu^4^, Yuanjun Zhu^2*^

^1^ Key Laboratory of Ecosystem Network Observation and Modeling, Institute of Geographical Sciences and Natural Resources Research, Chinese Academy of Sciences, Beijing 100101, China;

^2^ State Key Laboratory of Soil Erosion and Dryland Farming on the Loess Plateau, Northwest Agriculture & Forestry University, Yangling 712100, China;

^3^ College of Resources and Environment, University of Chinese Academy of Sciences, Beijing 100190, China;

^4^ Rothamsted Research, North Wyke, Okehampton, Devon EX20 2SB, UK

**
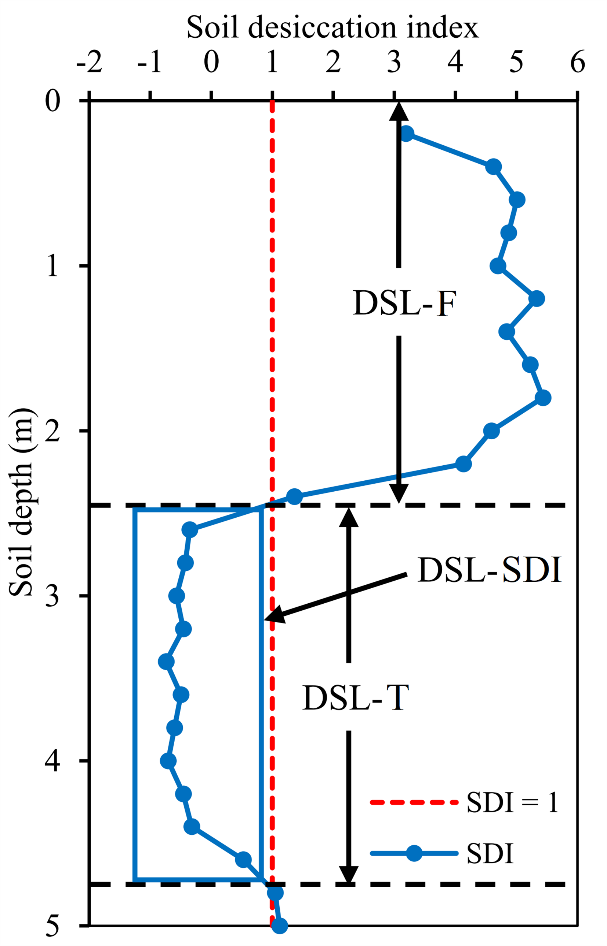
**

**Figure S1.** A plot of the three evaluation indexes of dry soil layer (DSL) of the soil proﬁle: The depth (DSL-F), thickness (DSL-T) and mean desiccation index (DSL-SDI) of DSL formation. Here, soil water data were collected in August 2013 at No. 38 sampling site along the transect.
